# Prevalence of left ventricular regional dysfunction differ in asymptomatic patients with systemic sclerosis compared to rheumatoid arthritis

**DOI:** 10.1186/1532-429X-13-S1-P318

**Published:** 2011-02-02

**Authors:** Kobayashi Yasuyuki, Joao AC Lima, Albert Lardo, Yasuo Nakajima, Hitomi Kobayashi

**Affiliations:** 1 St.Marianna University School of Medicine, Kawasaki, Japan; 2 Johns Hopkins University School of Medicine, Baltimore, MD, USA; 3 Itabashi Cuo General Hospital, Tokyo, Japan

## Background and purpose

In patients with systemic sclerosis (SSc) and rheumatoid arthritis (RA), cardiac involvements are common, may have serious consequences. Sensitive diagnostic techniques in cardiac MRI can identify subclinical myocardial abnormalities. The purpose of this study is to assess regional dysfunction of left ventricule in asymptomatic patients with SSc, compared to RA, by using unique image tracking method by using cine MRI.

## Methods

The study population included 21 patients, 11 patients with SSc and 10 patients with RA, who underwent evaluation with cardiac MRI. Peak systolic regional radial strain (Err, %) was calculated by image tracking method by using cine MRI based on the 16-segment model (ZIOsoft, CA, USA).

## Results

Compared with RA subjects, SSc patients had similar LV ejection fraction, but peak systolic Err was less negative in all of 6 segments calculated by 16-segment model in SSc than RA. Peak systolic Err is; 1) antero-septal wall: 0.38+/-0.18 in RA and 0.24+/-0.14 in SSc (r=0.22), 2) infero-septal wall: 0.44+/-0.23 in RA and 0.29+/-0.13 in SSc (r=0.22), 3) inferior wall: 0.56+/-0.19 in RA and 0.38+/-0.14 in SSc (r=0.08), 4) infero-lateral wall: 0.57+/-0.25 in RA and 0.52+/-0.20 in SSc (r=0.57), 5) antero-lateral wall: 0.51+/-0.19 in RA and 0.45+/-0.30 in SSc (r=0.47), 6) anterior wall: 0.67+/-0.28 in RA and 0.41+/-0.31 in SSc (r=0.17).

## Conclusions

SSc is associated with regional LV dysfunction in subclinical stage. Further large studies are needed to validate this finding and to better define implications of subclinical regional LV dysfunction.

